# Evaluation of a new secondary dose calculation software for Gamma Knife radiosurgery

**DOI:** 10.1002/acm2.12794

**Published:** 2020-01-14

**Authors:** Michael T. Prusator, Tianyu Zhao, James A. Kavanaugh, Lakshmi Santanam, Joe Dise, S. Murty Goddu, Timothy J. Mitchell, Jacqueline E. Zoberi, Taeho Kim, Sasa Mutic, Nels C. Knutson

**Affiliations:** ^1^ Department of Radiation Oncology Washington University School of Medicine in St. Louis St. Louis Missouri USA

**Keywords:** Gamma Knife, icon, secondary dose calculation, SRS

## Abstract

Current available secondary dose calculation software for Gamma Knife radiosurgery falls short in situations where the target is shallow in depth or when the patient is positioned with a gamma angle other than 90°. In this work, we evaluate a new secondary calculation software which utilizes an innovative method to handle nonstandard gamma angles and image thresholding to render the skull for dose calculation. 800 treatment targets previously treated with our GammaKnife Icon system were imported from our treatment planning system (GammaPlan 11.0.3) and a secondary dose calculation was conducted. The agreement between the new calculations and the TPS were recorded and compared to the original secondary dose calculation agreement with the TPS using a Wilcoxon Signed Rank Test. Further comparisons using a Mann‐Whitney test were made for targets treated at a 90° gamma angle against those treated with either a 70 or 110 gamma angle for both the new and commercial secondary dose calculation systems. Correlations between dose deviations from the treatment planning system against average target depth were evaluated using a Kendall’s Tau correlation test for both programs. The Wilcoxon Signed Rank Test indicated a significant difference in the agreement between the two secondary calculations and the TPS, with a *P*‐value < 0.0001. With respect to patients treated at nonstandard gamma angles, the new software was largely independent of patient setup, while the commercial software showed a significant dependence (*P*‐value < 0.0001). The new secondary dose calculation software showed a moderate correlation with calculation depth, while the commercial software showed a weak correlation (Tau = −.322 and Tau = −.217 respectively). Overall, the new secondary software has better agreement with the TPS than the commercially available secondary calculation software over a range of diverse treatment geometries.

## 1 INTRODUCTION

Gamma Knife (GK) radiosurgery has become a popular technique for the treatment of a variety of intracranial diseases, such as acoustic neuroma, pituitary adenoma, trigeminal neuralgia, vascular malformations, and malignant metastases.^1^, ^2^ Using 192 collimated Co‐60 sources focused at an isocenter, a patient will be stereotactically positioned to place the target at the source ray intersections to submillimeter accuracy.^3^ GK treatments are characterized by large doses delivered in a single, or more recently, hypofractionated schemes utilizing rigid thermoplastic masks with cone beam CT image guidance, and very sharp dose gradients outside of the target.^4^, ^5^ Because of the uniqueness of this system and treatments, quality assurance (QA) is of the utmost importance, including patient specific secondary dose calculations.^6^ Secondary independent dose calculations play an important role in radiation therapy, and given the high precision associated with GK radiosurgery, secondary dose checks become even more important to reduce the risk of doing serious harm to the patient.^7^, ^8^

Given the complicated geometry of GK treatments, establishing an accurate methodology to incorporate secondary dose calculations into the clinical workflow has been cumbersome.^8^ There have been several publications working to satisfy this clinical need, but many of the secondary dose calculation techniques suggested will still fail in certain situations, most notably when the patient is setup with a 110 or 70° gamma angle, or when the calculation point is at a shallow depth near the skull surface. It has been suggested that these difficulties in accurate secondary dose calculation arise from modeling the skull geometry, and constant density assumptions near the skull surface.^8^ Different skull rendering techniques have been proposed, including modeling the skull as a sphere or using measured skull data from the use of a skull scalar instrument.^8^, ^9^, ^10^ These methods work well for standard patient and target geometries, but significant discrepancies from the treatment planning system (TPS) are still evident when the target is at a shallow depth or when the patient setup uses a non 90° gamma angle.^11^ It has been suggested that using an image thresholding technique may be the best method to accurately construct the patients skull for dose calculation.^12^ Image thresholding makes use of an image data set such as CT or MRI and binarily assigns a voxel of the dataset to be within or beyond the skull boundary based upon a determined threshold image scale value. Skull rendering using this method minimizes uncertainties from measurement interpolation and produces an accurate representation of the true patient surface geometry.

Our institution installed the Leksell Gamma Knife Icon (Elekta Medical Systems, Stockholm, Sweden) in November of 2017. The Icon treatment system utilizes 192 Co‐60 sources divided into eight sectors that can be individually blocked, or collimated to 4, 8, and 16 mm shot sizes. The Icon is unique in that it utilizes on‐board cone beam computed tomography (CBCT) imaging system to enable fractionated and frameless treatments.^13^ The specifics on commissioning and QA for the Icon system can be found in the literature.^13^, ^14^, ^15^, ^16^ Dose is calculated for Icon patients using the TMR10 algorithm.^17^ This algorithm requires only dose rate calibration given by the user and a configurable collimator output factors that are provided by manufacturer. The TMR10 uses an exponential attenuation computation to the point of interest that is specific to each source location. The attenuation length (i.e. depth of the point of interest in the patient) for each source is calculated based on the source focal point, the distance from the focal point to the point of interest, and the distance to the rendered skull surface.^18^ For each GK Icon target treated, a secondary dose calculation using a commercially available software is completed prior to treatment per institution policy. This commercial secondary check software reportedly uses the same TMR10 dose calculation formalism as the TPS, and reconstructs the patient skull using 24 scalar measurements either input directly by the user or inferred from a CT dataset.^19^ The same user inputs were utilized for the second check software as the TPS. Our experience is similar to Xu et al.,^11^ especially where the commercially available software performs poorly in the presence of a nonstandard gamma angle, in some cases deviating from the TPS by more than 10%. This known issue presents a clinical difficulty in that treatment cannot proceed unless the TPS and secondary dose calculation agree to within 5%, as is the recommendation taken from AAPM Task Group 40 and our clinical policy.^7^ In many cases, the problem is circumvented by selecting a different point in the plan other than the point of maximum dose for comparison. Unfortunately, this limits the applicability of the integral purpose of independent secondary dose calculation.

In this work, a new secondary dose calculation engine based on the work of Mamalui‐Hunter et al.^12^ is evaluated and compared to a commercially available secondary check software. The new software reportedly uses the same dose calculation algorithm as described previously,^20^ but aims to solve the described deficiencies of previous secondary dose calculation methods. An image threshold skull rendering technique is utilized, and the gamma angle is precisely accounted for by applying a rotation matrix for each beamlet directly in the new software. Beamlets are created at each source position for each shot in each target. The beamlets themselves are then rotated and translated in space according to shot geometry, including gamma angle considerations. With the beamlets in their proper geometric positions about the skull, the vectors from source to isocenter and calculation point are ray traced through patient geometry for depth calculations to compute dose.

The combination of these two methods may result in more accurate calculation conditions for both standard and nonstandard gamma angles which in turn will provide a more robust secondary calculation engine that can be used in the clinical setting.

## 2 MATERIALS AND METHODS

To evaluate the new secondary dose calculation software (RadCalc, Lifeline Software, Austin, TX) after the review and approval from the Institutional Review Board (HRPO# 201904138), the first 800 targets treated since the Icon’s installation at our institution were exported from our treatment planning software (TPS) (GammaPlan 11.0.3, Elekta Medical Systems, Stockholm, Sweden) to the new secondary calculation patient database. These 800 targets were a sufficient representative of the patient population that is typically treated at our institution. Target positions ranged from the periphery of the skull to more central locations. This resulted in a wide range of average calculation depths, from 2.4 to 10.5 cm. The target population used in this study also included a variety of non‐standard patient setup geometries where a gamma angle of 70 or 110° was utilized (30 cases).

**Figure 1:**
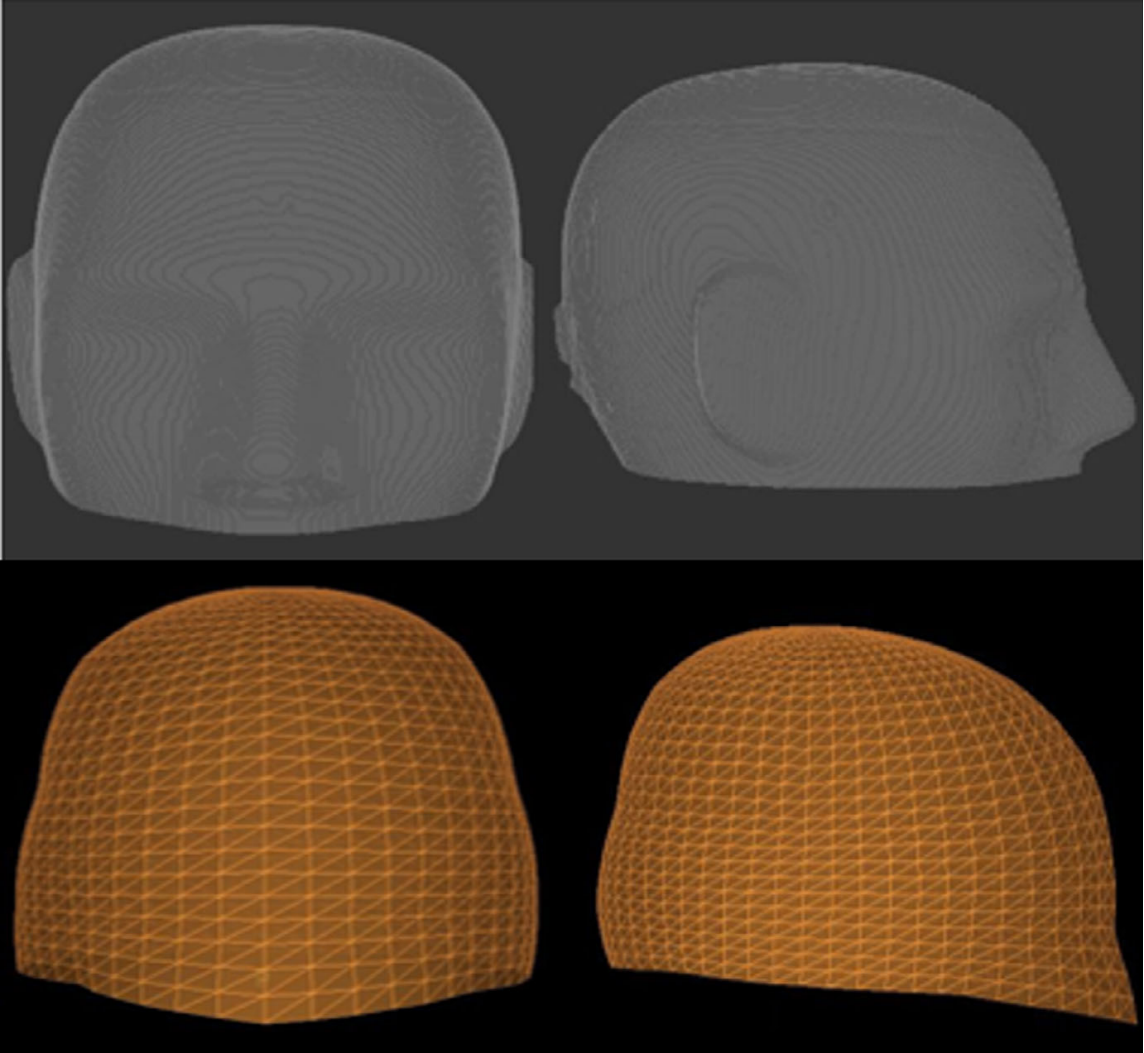
Top: A reconstructed phantom skull surface geometry using the image thresholding technique from the new secondary calculation software for a head phantom. Bottom: A reconstructed phantom skull surface geometry using scalar measurements from the commercial software.

After each target was imported into the new secondary calculation software, the patient skulls were constructed using the image thresholding technique, in contrast with the derived scalar measurements used in the commercial software (Gamma Check, MU Check, Oklahoma City, OK), available with the program (Fig. 1). In most cases, the skulls were rendered from a CT image dataset. However, in approximately 10% of the patient plans a CT dataset was not available and the patient’s skull was rendered using an MRI dataset. The current versions of the TPS support image thresholding skull definition from both types of imaging modalities. Individual beamlet rotations were also made for patient setups using non‐standard gamma angles. Dose was re‐calculated with the new software, and the agreement to the TPS was evaluated. This was done using the percent difference for each individual target. The median and range of the percent differences per target was calculated over the entire cohort. A further comparison of deviation between TPS and secondary calculation doses with respect to average calculation depth was completed using a Kendall’s Tau correlation test to evaluate any dependencies the secondary software has on target depth in the skull. The target cohort was then binarily categorized by gamma angle (standard 90° and nonstandard 70/110°). Using a Mann‐Whitney U test, differences between the two categories with respect to agreement with the TPS were evaluated for statistical significance.

A Wilcoxon Signed Rank Test (WSRT) was used to evaluate significant differences in the deviation from the TPS calculated dose over the entire 800 treatment targets for both calculation software packages. Using the same techniques as described for the new secondary dose calculation software, dependencies on agreement to the TPS of the commercially available software dose calculation as it pertains to gamma angle and average calculation depth were evaluated and compared to the dependencies of the new secondary dose calculation software.

The new secondary dose calculation software has the capability to render the skull for dose calculation using discrete scalar measurements if these measurements were used in the TPS for planning. To isolate the robustness of the beamlet rotation algorithm employed for nonstandard gamma angles by the new software, the target with a nonstandard gamma angle patient setup that showed the largest discrepancy from the TPS by the commercially available software was replanned in GammaPlan using skull scalar measurements. The plan was reimported to the new software and the skull was rendered using the discrete measurements. Dose was recalculated using the new software and compared with the TPS.

As the purpose for a secondary dose calculation is to give the user confidence that the primary dose calculation from the TPS is accurate, an absolute dose measurement was also employed for comparison against both the TPS and the two secondary dose calculation engines. In the two plans where the TPS estimated dose and the commercially available secondary dose software differed the greatest, the shot arrangement and gamma angle in the patient plan was copied to an anthropomorphic phantom and a film measurement was taken. Using EBT3 gafchrormic film cross calibrated to an ADCL calibrated ionization chamber, an optical density to dose calibration curve was created by irradiating a 16 mm shot to 2, 3, 4 and 5 Gy. Next, the patient plan was scaled to a max dose of 4 Gy, and delivered on an anthropomorphic head phantom. The maximum measured dose was then compared to the TPS, a calculation from commercially available software, and what the new secondary dose calculation software predicted. Each of the film measurements, including calibration films, were repeated three times.

## 3 RESULTS

**Figure 2:**
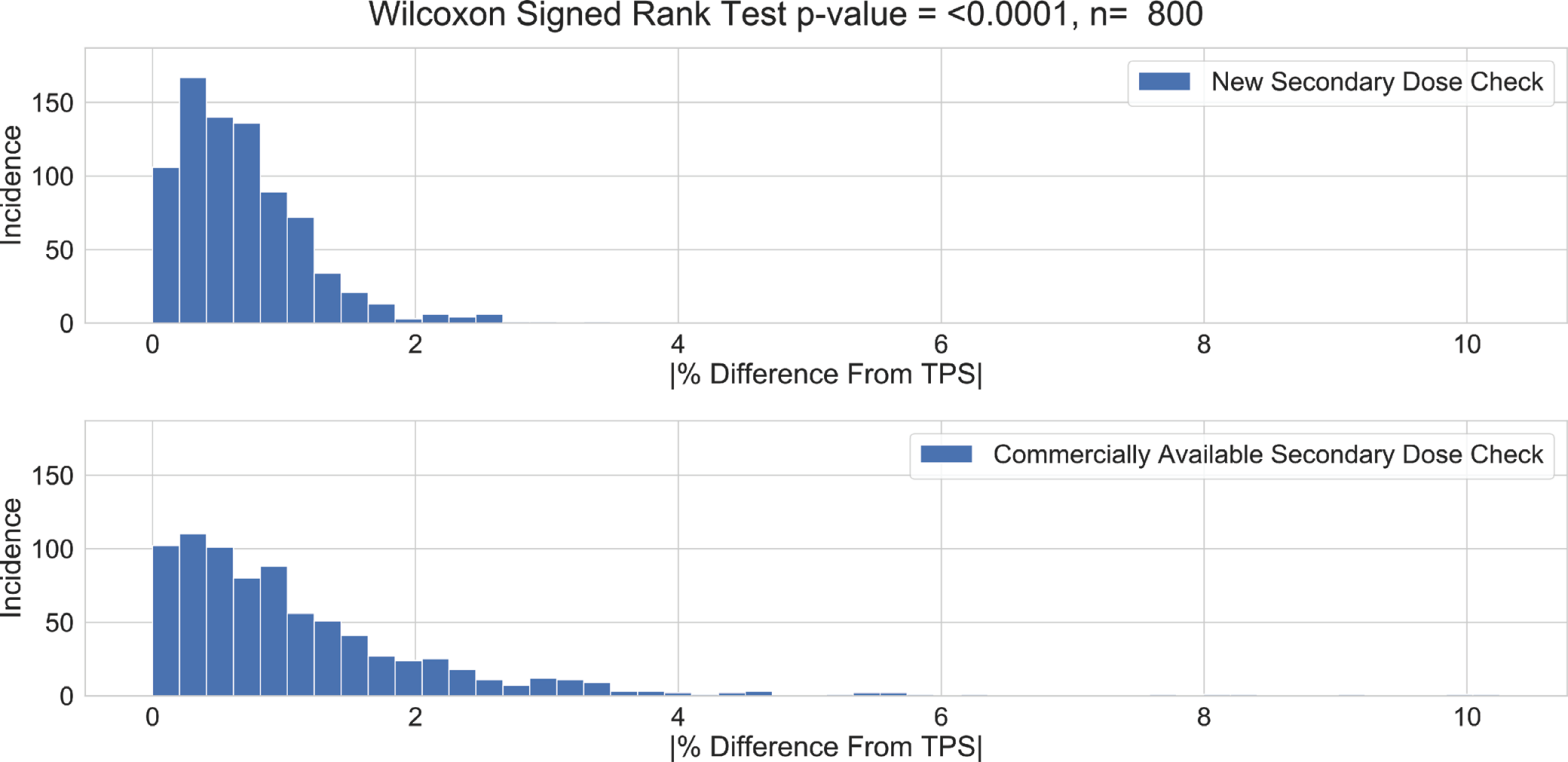
The absolute percent differences from the TPS for each of the 800 targets calculated using the new secondary dose check software and the commercially available secondary dose calculation software. TPS, treatment planning system.

**Figure 3:**
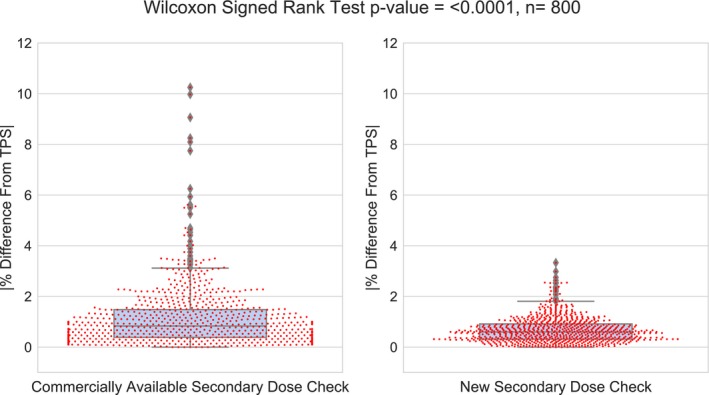
A box and swarm plot of the absolute differences from the TPS and the Wilcoxon Signed Rank Test for each of the 800 targets using the commercial and new secondary dose calculation software. TPS, treatment planning system.

Over the entire target cohort, the new secondary dose calculation software showed excellent agreement with the TPS. The differences from the TPS ranged from 0.00% to 3.33%, with a median and mean value of 0.6% and 0.68%, respectively, which is well within our clinical tolerance. The agreement between the TPS and the commercially available dose calculation software showed larger deviations, ranging from 0.00% to 10.25% and a median and mean value of 0.833% and 1.15%, with 13 above clinical tolerance. These data are shown in Fig. 2. The WSRT revealed a significant difference in the agreement deviations between the new and commercially available secondary dose calculations, with a *P*‐value < 0.0001, where the overall agreement between the new secondary dose calculation software and the TPS was superior as shown in Fig. 3.

**Figure 4:**
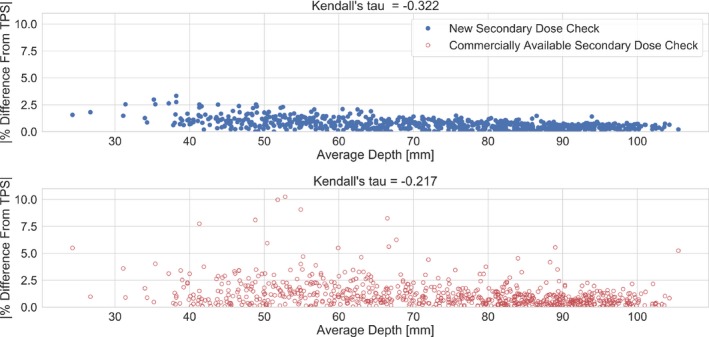
The results of the Kendall’s tau correlation evaluation. The absolute percent difference from the TPS for the new software shows a moderate correlation with average calculation depth (tau = −0.322). The absolute percent difference from the TPS for the commercial software shows a weak correlation with average calculation depth (tau = −0.217). TPS, treatment planning system.

With respect to the secondary dose calculation dependency on average calculation depth, the Kendall’s tau correlation test showed that a moderate (tau = −.322), and weak (tau = −.217) inverse correlation exists between calculated dose discrepancy from the TPS and calculation depth for the new secondary dose calculation software and the commercially available software, respectively, shown in Fig. 4. This suggests that for the new secondary dose calculation software, the discrepancy in calculated dose from the TPS may increase slightly for targets at shallower depth. However, for the commercial software the depth does not seem to correlate with percent difference.

**Figure 5:**
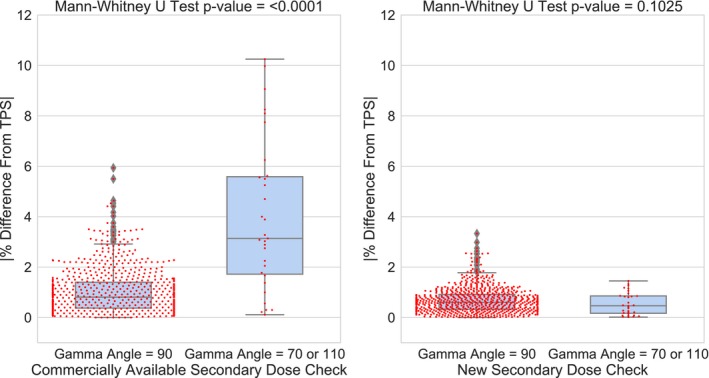
The comparison of percent differences with regards to standard and non‐standard gamma angle setups for both the commercial software and the new software. The new software does not show a significant dependency on gamma angle in patient setup (*P* = 0.1025).

The gamma angle does not appear to play a large role in the calculated dose agreement between the new secondary dose calculation software and the TPS. When compared to dose deviations from the TPS at a gamma angle of 90°, the discrepancies from the TPS at gamma angles of 70/110° were not significant (*P*‐value = 0.102). This is in contrast with what was seen with the commercially available calculation software, where the discrepancies between dose calculations were significant for standard vs nonstandard gamma angles (*P* < 0.0001), shown in Fig. 5. When looking at dose deviations for gamma angles of 90° only, the new secondary calculation software showed significantly smaller deviations from the TPS when compared to the commercial software (*P* < 0.0001).

The largest deviation from the TPS calculated dose using the commercially available secondary software was 10.25%. This particular plan had a nonstandard gamma angle of 70°. The corresponding point with the new check software using image threshold skull rendering was 1.3 percent. The new secondary calculation software has the capability to construct the skull for calculation using scalar measurements, assuming the skull was constructed this way in the TPS. To isolate how the two secondary calculation engines compare to the TPS when both use scalar measurements for skull rendering, this plan was recalculated in Gamma Plan with skull scalar measurements (as opposed to an image dataset) and a secondary dose check was repeated using both systems. When recalculated in the new software using skull scalar measurements the agreement remained within clinical tolerance at 0.1%, the corresponding agreement using the commercial software was still above tolerance at 6.5%.

In the two cases where the commercial secondary calculation and the TPS differed the greatest, one target utilized a gamma angle of 70° in the plan, while the other had a gamma angle of 110°. When these target plans were replanned on the anthropomorphic phantom to a maximum dose of 4 Gy, the commercial and new secondary dose calculated a max dose of 4.36 Gy (9.2% higher than the TPS) and 4.047 Gy (1.2% higher than the TPS) for the 70° gamma angle plan and 4.39 Gy (9.1% higher than the TPS) and 4.044 Gy (1.1% higher than the TPS) for the 110° gamma angle plan. However, the film measurement resulted in a delivered dose of 3.91 Gy +/− 0.04 Gy to the 70° gamma angle plan differing from the TPS by −2.25%, and 3.88 Gy +/− 0.08 Gy to the 110° plan, differing from the TPS by −3%. Both the TPS and new secondary dose calculation fall well within our clinic’s 5% agreement criteria with measurement, while the commercial secondary calculation falls well outside of the measurement results.

## 4 DISCUSSION

The results from this study show promising clinical applicability for the new secondary dose calculation software. Each of the 800 targets calculated are well within our clinical tolerance of 5%. The distribution of deviations from the TPS between the commercial software and the new calculation software show a significant difference, with the deviations being smaller for the new software. This shows strong evidence that the new secondary dose calculation software is a robust platform for independent dose calculation that effectively handles complex patient setup and geometries. This is especially true when considering the use of nonstandard gamma angles for patient positioning. The commercially available software falls short in several instances where a gamma angle of 70 or 110° is used. In fact, the Mann‐Whitney test shows a significant difference in the deviations from the TPS between standard and nonstandard gamma angles for the commercially available secondary dose calculation software, confirming dependency on the patient orientation that has been previously shown in literature.^11^ However, the same comparison is not significant for the new secondary dose calculation software, indicating that the method of beamlet rotation matrices eliminates this dependence. This argument is further strengthened by the skull scalar measurement example using the new calculation software, as well as by the absolute film dose measurement. In skull scalar measurement example, both the commercially available and secondary dose software rendered the skull using skull scalar measurements, removing the dependency on the rendering of the skull and isolating the methods used by these two programs to handle gamma rotations. The new software calculation was still well within tolerance, while the commercial software showed a discrepancy of 6.5%. With regards to measurement, for the cases where the commercial software disagrees significantly from the TPS, the new software does agree well within tolerance to the TPS and absolute film dose measurement. This suggests that while the new software agrees well with the TPS, it is also independently accurate in calculating dose for complex treatment plans where nonstandard gamma angles are used in patient setup.

**Figure 6:**
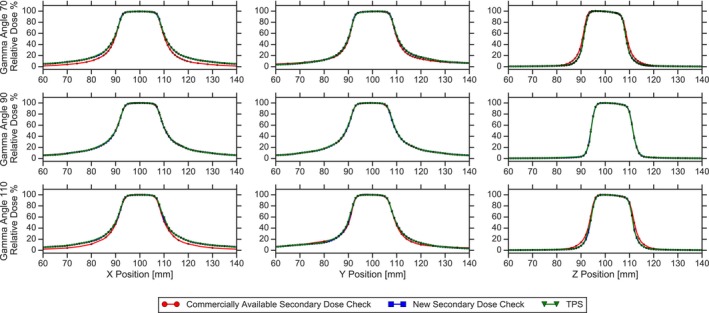
A comparison of the dose profiles for a single 16mm shot calculated using the TPS, the commercial software and the new secondary dose calculation software. From left to right, the profiles are taken in X, Y and Z planes, respectively. From superior to inferior, the profiles are at gamma angles of 70, 90° and 110°, respectively. For all gamma angles, the new dose calculation software and the TPS match well. However, at gamma angles of 70 and 110°, the commercial software show discrepancies in the tail and penumbra regions. TPS, treatment planning system.

The gamma rotation technique of the commercial software is relatively unclear. The information on this topic available to the user via vendor provided documentation provides a short summary for the Gamma Knife model 4C, but gives little insight for the Icon.^19^ Based upon this documentation it appears that for the 4C, the general strategy of translating source position with respect to gamma angle is similar to the new software. However, the implementations of the beamlet translation algorithm for the Icon used by the two different secondary calculation engines are not equivalent. This is apparent when looking at the profiles of each 16 mm shot in the X, Y, and Z directions for each gamma angle. Figure 6 shows a comparison of the dose profiles taken from the TPS, the commercial software, and the new software in each plane and for gamma angles of 70, 90, and 110°. When looking at the comparison for 90° gamma angle, the agreement between the three dose calculations is exceptional. However, at the nonstandard gamma angles, there are large discrepancies in the penumbra and tail regions of the profiles. In the X direction, the commercial software significantly underpredicts the dose when compared to the new software and the TPS. In the Y direction, the commercial profile is asymmetric compared to the other two calculations, where one side of the profile matches reasonably well, and the opposite side calculates a lower dose in comparison. In the Z direction, the commercial software overpredicts dose compared to the other two calculation algorithms. However, in all three planes for each gamma angle setup, the profiles of the new software and the TPS agree well. It is likely that for this reason large discrepancies from the TPS dose calculations are seen in the commercial software but not the new secondary dose calculation software. When two or more shots are in close proximity with nonstandard gamma angles, the propagation of errors in the tail regions of the profiles compounding on the high dose area of another shot will cause a large disagreement between the two calculations that is not seen with the new secondary dose calculation software.

Looking at the calculations for patients treated at 90° gamma angles only, the new secondary software had significantly (*P* < 0.0001) smaller deviations from the TPS than the commercial software. This indicates that the imaging threshold method of skull rending out performs scalar measurements with interpolation, suggesting that a patient’s head may not always be well‐described by 24 scalar measurements. Rendering the skull using this image thresholding technique gives much more flexibility for accurate calculation over a wide range of skull shapes and sizes.

While the new software showed excellent agreement with the TPS and measurement, the calculation deviation does appear to have a small dependence on the average calculation depth, as shown by the moderate correlation in the Kendall's tau test. It is not obvious as to what the source of this dependency is, but it could be a function of interpolation in the 3D computation matrix to a single point dose that is more evident at shallower depths. However, in the commercial software, depth showed a weak correlation to the agreement with the TPS. This result did not support the previous suggestions in the literature that the depth of the target is a driving factor in the accuracy of the secondary dose calculation. However, this work did show a strong dependency for the commercial software on the gamma angle as suggested in the literature.

## 5 CONCLUSION

Currently, secondary dose calculation in Gamma Knife radiosurgery is not robust and current methods of computing dose accurately depend on the simplicity of treatment geometry. In complex patient setups, these current secondary calculation methods fail, leading the user to make difficult clinical decisions on whether to proceed with treatment that may not be warranted due to the inaccuracy of secondary dose calculation. In this study, a new secondary dose calculation software for Gamma Knife radiosurgery using image threshold skull rendering and beamlet rotation technique was evaluated and compared to a commercially available software for 800 targets treated with our Icon system. The new software clearly excels where the commercial software falls short, especially in the presence of 110° and 70° gamma angles in patient setup. This will make a large impact for the Gamma Knife physicist for plan QA by providing confidence to the user that the planned dose calculated by the TPS is accurate, regardless of complexity of calculation geometry.

## CONFLICT OF INTEREST

The department of radiation oncology physics division received funding in aiding the implementation of previous work from our department into a secondary dose calculation program for Gamma Knife from Lifeline Software Inc.
